# *Coccidioides* genomes from low-incidence states reveal complex migration history across the western United States

**DOI:** 10.1128/spectrum.01822-25

**Published:** 2025-10-24

**Authors:** Emanuel M. Fonseca, Shanaya Fox, Adrienne L. Carey, Bridget Barker, Marco Marchetti, Megan Hirschi, Kimberly E. Hanson, Katharine S. Walter

**Affiliations:** 1 Division of Epidemiology, University of Utah, Salt Lake City, Utah, USA; 2 Elfa Analytics, Ponte Nova, Minas Gerais, Brazil; 3 Department of Medicine, Division of Infectious Diseases, University of Utah School of Medicine, Salt Lake City, Utah, USA; 4 Department of Biological Sciences, Northern Arizona University, Flagstaff, Arizona, USA; 5 Eccles Institute of Human Genetics, University of Utah, Salt Lake City, Utah, USA; 6 Department of Pathology, Division of Clinical Microbiology, University of Utah and ARUP Laboratories, Salt Lake City, Utah, USA; Institut Pasteur, Paris, France

**Keywords:** *Coccidioides*, coccidioidomycosis, genomic epidemiology, phylogenetics, fungal pathogen, disease surveillance

## Abstract

Coccidioidomycosis (Valley fever), caused by the fungal pathogen *Coccidioides*, is increasing in incidence across the western United States and parts of the Americas. However, genomic data from low-incidence regions remain scarce, limiting our understanding of the pathogen’s dispersal and evolution. To address this gap, we prospectively collected *Coccidioides*-positive clinical isolates submitted to a national diagnostic laboratory between January 2023 and November 2024. We performed whole-genome sequencing on 27 clinical isolates from Utah, Colorado, and Nevada—states with no previously available *Coccidioides* genomes—and sequenced an additional 22 isolates from California. For Utah patients, we also reviewed medical records to assess potential travel history. Among the 27 newly sequenced isolates, three were identified as *Coccidioides immitis* and 24 as *Coccidioides posadasii*. The *C. immitis* isolates, all from a single Utah patient, were linked to recent travel to southern California. In contrast, *C. posadasii* isolates from Utah, Colorado, and Nevada were phylogenetically diverse and dispersed across the species tree. Ancestral state reconstructions suggested multiple independent introductions: at least seven into Utah, two into Nevada, and seven into Colorado. The *C. immitis* clade likely originated in California (likelihood = 0.96), while *C. posadasii* traces to Arizona (likelihood = 0.99). These results reveal a complex dispersal history of *Coccidioides* in the western United States, driven by recurrent introductions rather than a single emergence event. Our study underscores the need for continued genomic surveillance in underrepresented regions to fully capture the evolutionary dynamics of this emerging fungal pathogen.

**IMPORTANCE:**

Valley fever is a fungal disease caused by *Coccidioides* species, primarily found in arid regions of the western United States and parts of Central and South America. While most genomic studies focus on high-incidence areas, the evolutionary dynamics of the fungus in low-incidence states remain poorly understood. In this study, we analyzed clinical *Coccidioides* isolates from Utah, Colorado, and Nevada—states with reported cases but limited genomic data. Using whole-genome sequencing and phylogenetic analysis, we found evidence of multiple introductions into each state, likely from neighboring high-incidence regions such as Arizona and California. In Utah, we detected both *Coccidioides immitis* and *Coccidioides posadasii*, though the *C. immitis* case was associated with recent travel. Only *C. posadasii* was found in Colorado and Nevada. These findings support ongoing dispersal rather than a single introduction and highlight the need for expanded genomic surveillance beyond traditionally endemic regions.

## INTRODUCTION

*Coccidioides immitis* and *Coccidioides posadasii* are soil-dwelling fungal pathogens that cause coccidioidomycosis, commonly known as Valley fever. This disease, which ranges from mild respiratory illness to severe systemic infection, results from the inhalation of airborne arthroconidia produced by these fungi (1, 2). Endemic to arid and semi-arid regions of the Americas, the two species exhibit distinct geographic distributions. *C. immitis* is primarily found in California and Baja Mexico, though recent evidence suggests its range is expanding, with newly identified isolates in Washington and Oregon (3, 4). In contrast, *C. posadasii* spans a broader area, including the southwestern United States (Arizona, Texas, and New Mexico), Mexico, and parts of Central and South America (3, 4). The ecological niche of these fungi is closely linked to specific environmental conditions—warm temperatures, seasonal rainfall followed by dry periods, and elevated dust levels—that promote their growth and dispersal (5, 6).

Coccidioidomycosis is increasingly recognized as a re-emerging infectious disease, with rising incidence rates in endemic regions and an expanding geographic footprint (7–9). Recent modeling work has found that the expansion of reported cases of coccidioidomycosis is driven by climate change and land use alterations (6, 10). For instance, warmer temperatures and prolonged droughts may enhance fungal growth, while urbanization and agricultural activities disturb soil, releasing spores into the air. Despite its growing public health significance, coccidioidomycosis remains underdiagnosed and underreported due to its nonspecific symptoms and limited awareness among some healthcare providers (11, 12). As the distribution of *Coccidioides* continues to extend beyond historically endemic areas, there is an urgent need to better understand its evolutionary history and historic migration patterns of *Coccidioides* to understand ongoing and future dispersal.

While extensive genomic research has focused on high-incidence endemic regions (e.g., 4, 13, 14), low-incidence states such as Utah, Colorado, and Nevada remain poorly characterized in terms of genomics, despite documented cases of coccidioidomycosis. Due to the lack of genomic data from these regions, it remains unclear which *Coccidioides* species are locally endemic. For example, one study reported that both species were present at a single sampling site in Dinosaur National Park, in northeastern Utah (8). Further, it limits our ability to reconstruct migration patterns and identify potential geographic reservoirs for the fungus, which is responsible for seeding introductions elsewhere.

Here, we characterized the evolutionary history of *Coccidioides* in low-incidence states. We prospectively collected and, for the first time, sequenced clinical isolates from Utah, Colorado, and Nevada; for the Utah isolates, we also obtained detailed travel histories. We combined a *Coccidioides* phylogeny with information on the location of Valley fever patients to reconstruct historic patterns of *Coccidioides* spread and measured the number of independent introductions into each state.

## RESULTS

### Species distribution across endemic and low-incidence states

**Fig 1:**
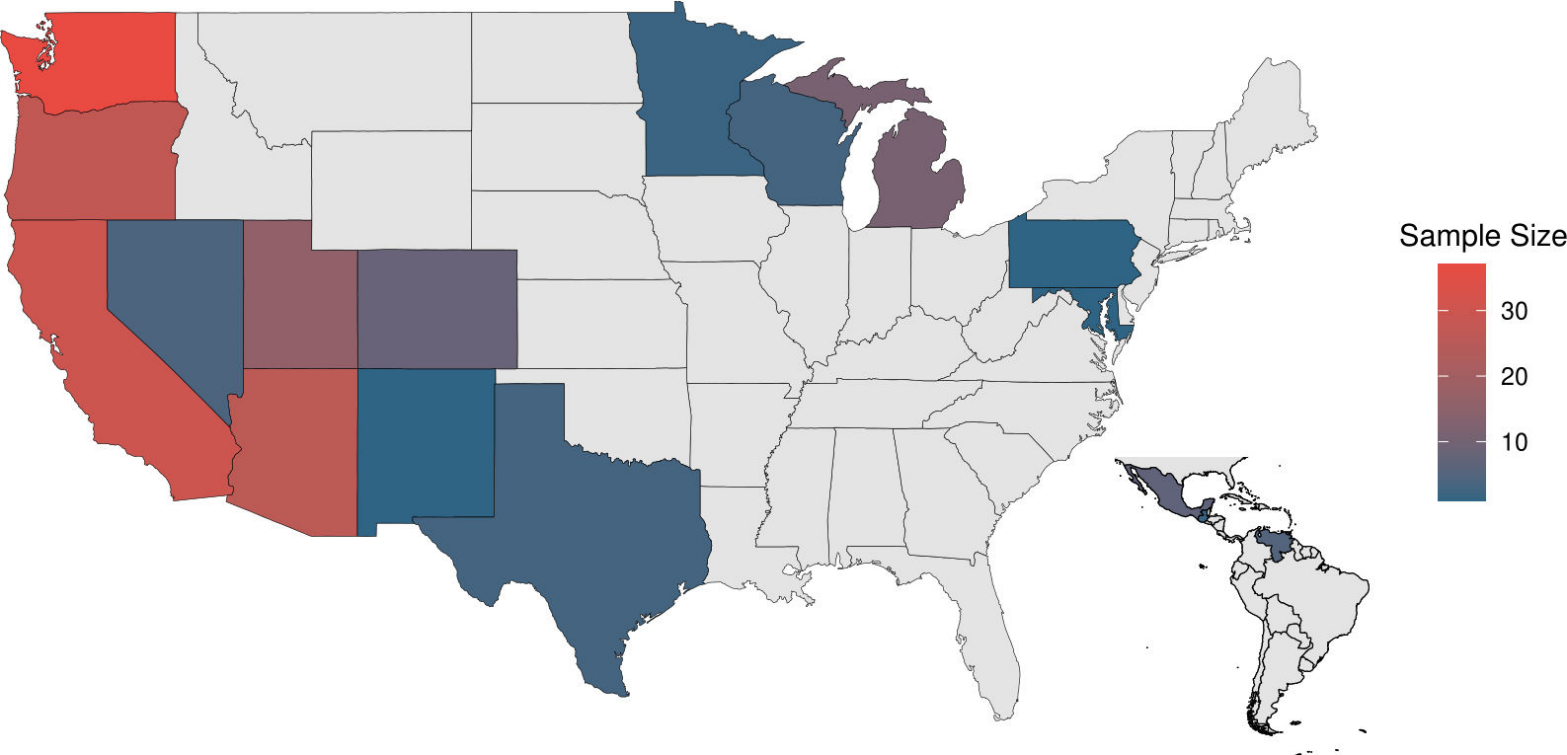
Sample sizes of sequenced *Coccidioides* genomes from across the United States, represented by a gradient color scale from blue (smallest sample size) to red (largest sample size). The inset map highlights samples collected outside the United States.

**Fig 2:**
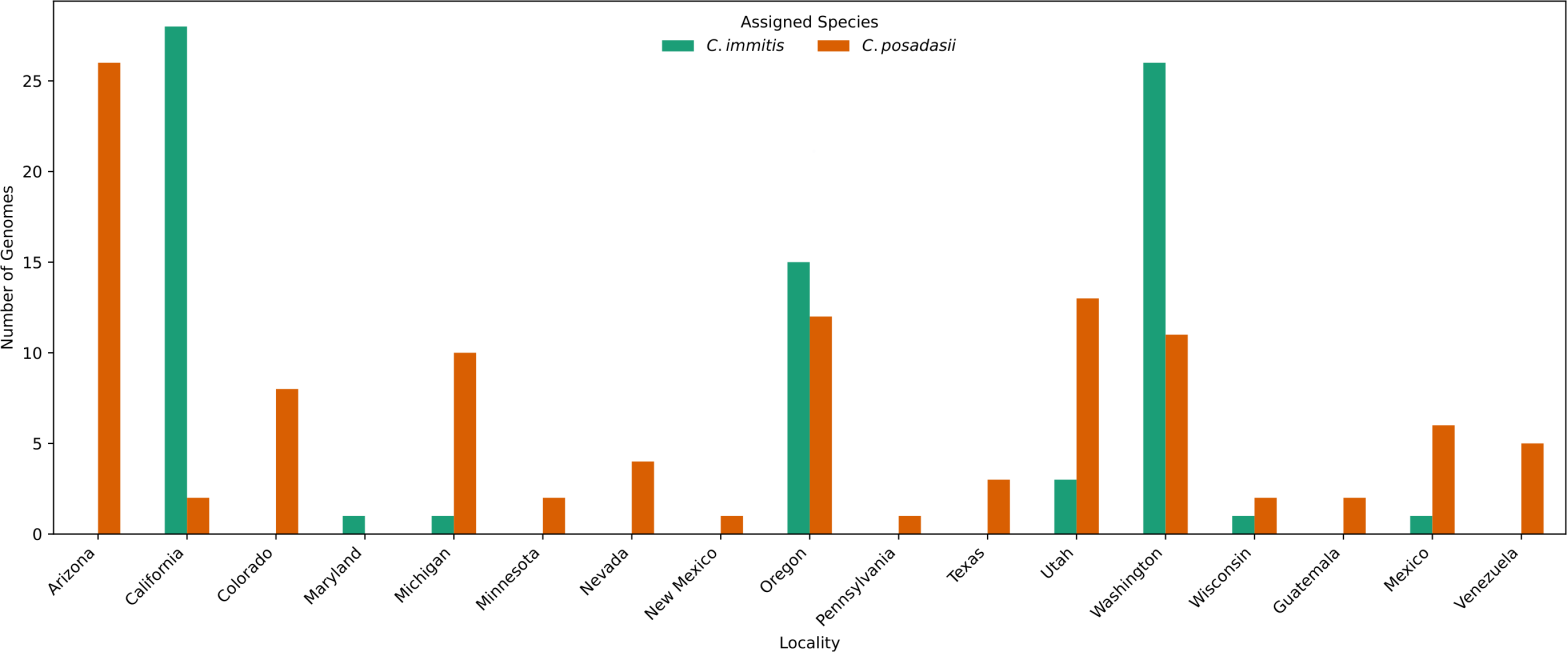
Species identification across sampled localities. Bars represent the number of samples per locality, with *Coccidioides immitis* shown in blue and *Coccidioides posadasii* in orange. The *x*-axis denotes sampled localities, while the *y*-axis indicates the corresponding sample count. The figure includes both newly sequenced samples collected through ARUP and previously published genomes obtained from public databases.

Among the 27 newly sequenced isolates from low-incidence states (Utah, Colorado, and Nevada) (Fig. 1), 24 were identified as *C. posadasii*, while only 3 were *C. immitis*. Notably, Utah was the only low-endemicity state where both species co-occurred, with *C. immitis* detected in 3 isolates from a single patient and *C. posadasii* found in nine (Fig. 2). In contrast, *C. posadasii* was the sole species detected in Colorado and Nevada (Fig. 2). In high-endemicity states, *C. immitis* was predominant in California, where 20 out of 22 newly sequenced isolates belonged to this species. In contrast, *C. posadasii* was more prevalent in Arizona, as reflected by the broader sample representation in this region, including the two remaining sequenced isolates from California (Fig. 2).

### Epidemiological profiles and exposure histories of Utah patients

We conducted a medical chart review for 4 of the 10 Utah patients who ranged in age from 41 to 70 years and included three women and one man. Among them, one individual reported a travel history to San Diego and Alaska and had previously lived in southern California. This individual was infected with *C. immitis* and was likely exposed outside of Utah. Clinical data, such as the timing of symptom onset or disease presentation, were not available, which limits our ability to more precisely evaluate the exposure window. The other Utah patients with available chart reviews had no recorded travel history.

The nine other Utah patients were infected with *C. posadasii*, suggesting that this species is the only endemic species in Utah.

### Phylogenetic evidence for multiple independent introductions into low-incidence states

**Fig 3:**
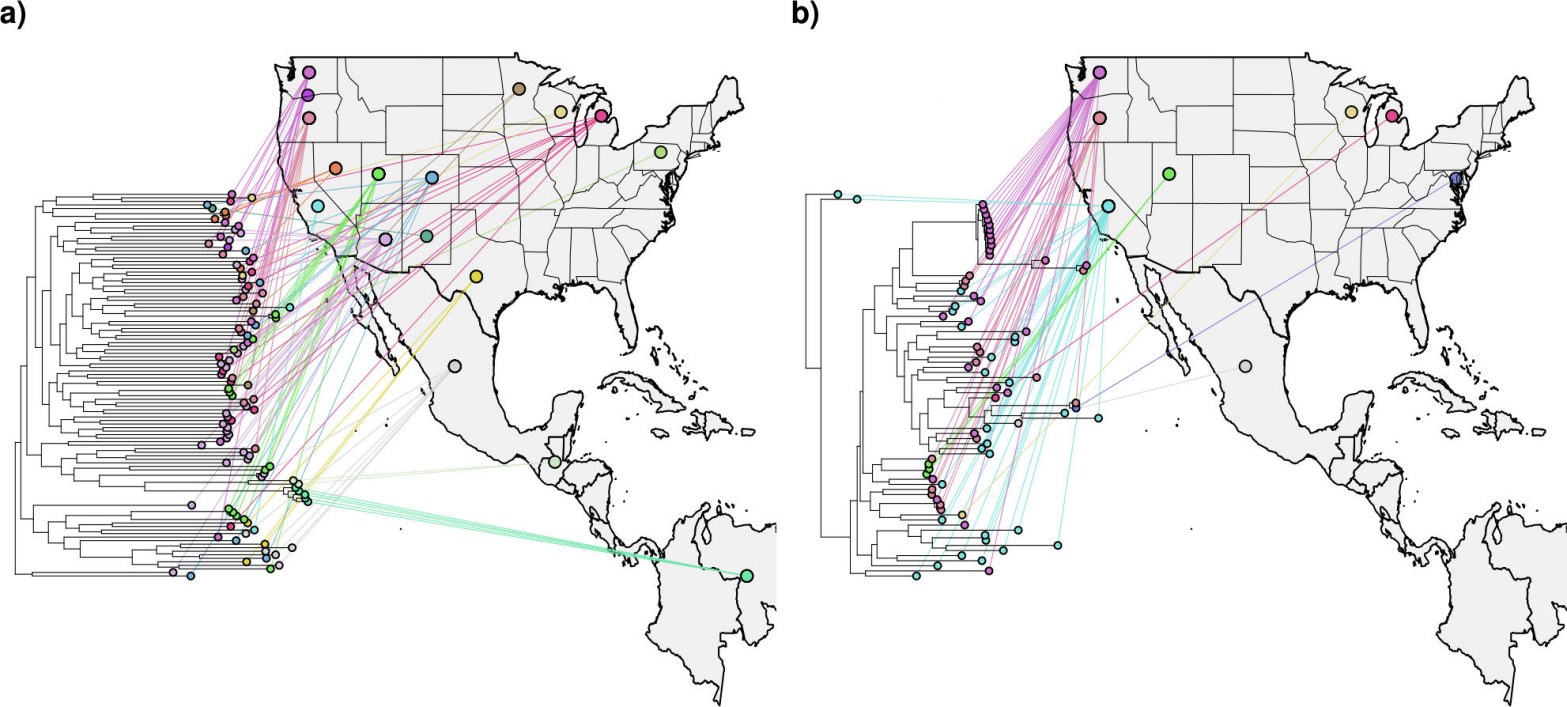
Maximum likelihood phylogenies illustrating the evolutionary relationships among sampled isolates of *Coccidioides*, with panel (**a**) showing *Coccidioides posadasii* and panel (**b**) showing *Coccidioides immitis*. The accompanying maps indicate the US state and country of origin for each isolate, highlighting patterns of geographic distribution and phylogenetic clustering.

In a maximum likelihood phylogeny of 185 *Coccidioides* samples, isolates from Utah, Colorado, and Nevada did not form monophyletic state-specific clades but were instead distributed across multiple lineages (Fig. 3A and B). This pattern suggests multiple independent introductions into low-endemicity states rather than a single expansion event (Fig. 3A). Phylogenetic analysis supports this observation, as geographically proximate isolates did not group strictly according to state boundaries. Additionally, the single Utah isolate of *C. immitis*, obtained from one patient, likely represents an infection acquired through travel, as the patient had documented travel history to San Diego and previously resided in Southern California. While travel histories are not available for all cases, our focus is on low-incidence states for which we do have partial epidemiological information. Importantly, we clarify that infections identified outside the known endemic region are presumed to be travel-acquired.

### Inference of geographic origins and dispersal pathways using ancestral area reconstruction

**Fig 4:**
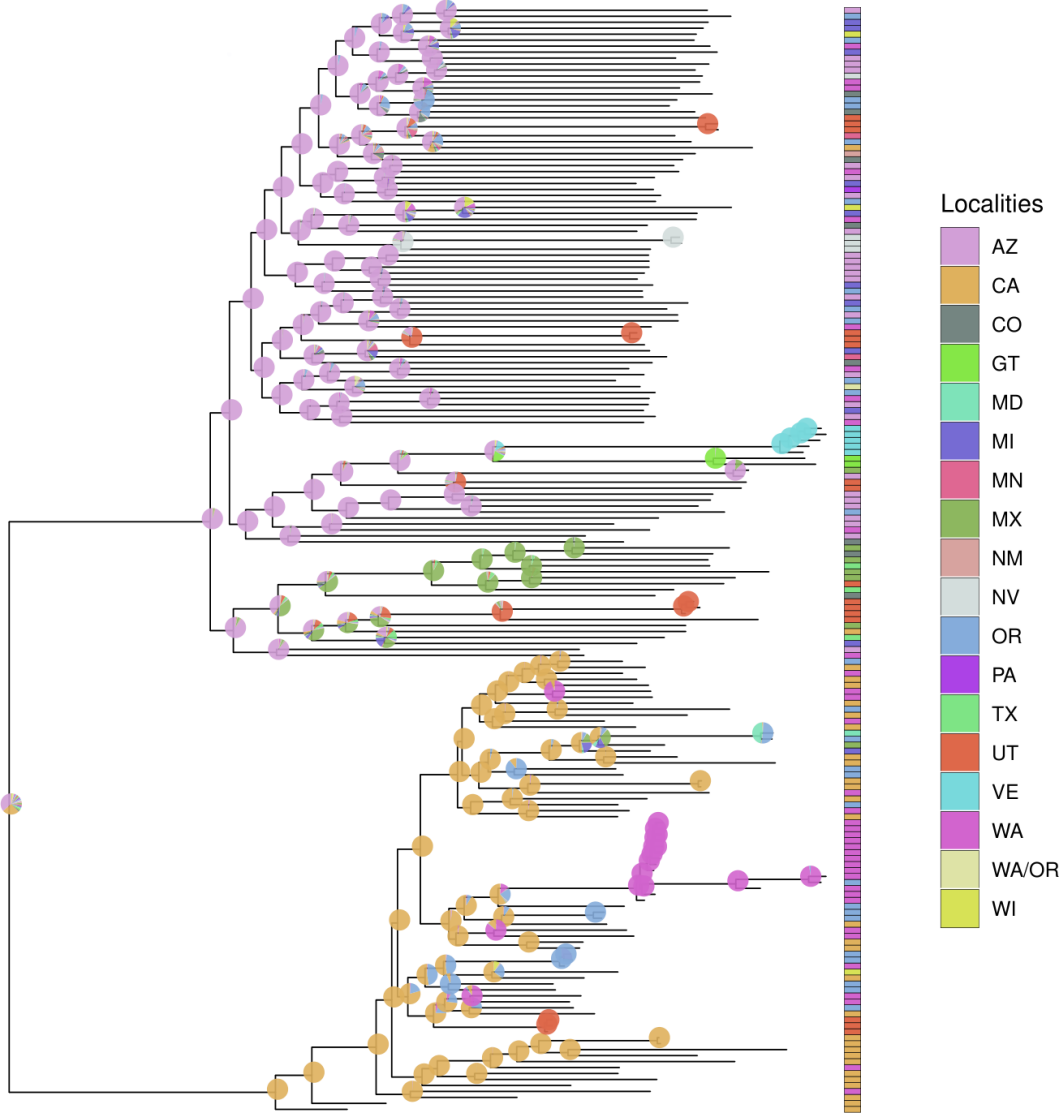
Maximum likelihood phylogeny illustrating ancestral area reconstruction across sampled isolates. Pie graphs on internal nodes represent the probability the ancestor occurred at a specific location, with possible localities including Arizona (AZ), California (CA), Colorado (CO), Guatemala (GT), Maryland (MD), Michigan (MI), Minnesota (MN), Mexico (MX), New Mexico (NM), Nevada (NV), Oregon (OR), Pennsylvania (PA), Texas (TX), Utah (UT), Venezuela (VE), Washington (WA), a combined Washington/Oregon (WA/OR) category, and Wisconsin (WI). Branch lengths represent genetic divergence, measured as the number of substitutions per site.

Ancestral area reconstruction revealed multiple independent introductions of *C. posadasii* into Utah, Nevada, and Colorado (Fig. 4). Utah received at least seven introductions, primarily originating from Arizona (estimated probability: 50–60%), with secondary contributions from Texas (20–30%) and California (10–20%). Nevada experienced two introductions, most likely from California (70–80%), with a smaller contribution from Arizona (20–30%). Colorado also had seven introductions, predominantly sourced from Arizona (40–50%) and Texas (30–40%), with additional input from New Mexico (10–20%). Minor introductions from other endemic regions, such as Mexico or Utah, were detected but occurred with lower probability (probabilities < 10%). These patterns highlight the role of neighboring endemic areas, particularly Arizona, Texas, and California, in seeding *C. posadasii* populations in low-incidence states.

The common ancestor of sampled *C. posadasii* isolates was likely in Arizona, with a probability of 96%, while the common ancestor of sampled *C. immitis* isolates was likely in California, with a probability of 99%. However, the common ancestor of both sister species could not be determined with high probability, with estimates suggesting either California (30%) or Arizona (25%) as possible ancestral locations. The three *C. immitis* isolates from Utah, all originating from the same patient, appear to represent a travel-associated introduction from California, consistent with the patient’s documented travel history to San Diego and prior residence in Southern California.

Although clinical data, such as disease presentation and timing of symptom onset, are not available, the travel history supports the interpretation that exposure occurred outside Utah. All three isolates were collected on the same day, which limits the opportunity to assess within-host genomic changes over time.

## DISCUSSION

The genetic and evolutionary history of *C. immitis* and *C. posadasii* has been previously studied in endemic regions (e.g., references 4, 13, 14); however, their presence in low-incidence states remains poorly characterized. Our study sought to bridge this gap by analyzing newly sequenced isolates from Utah, Colorado, and Nevada. Among the 27 newly sequenced clinical isolates, 3 were identified as *C. immitis* and 24 as *C. posadasii*. Utah was the only low-endemicity state where both species were detected, with *C. posadasii* identified in nine patients and *C. immitis* in a single individual. Although this case was likely travel-associated, given the patient’s prior residence in California, the environmental presence of *C. immitis* in Utah has been previously documented. Soil samples collected from Dinosaur National Monument in 2006 confirmed the co-occurrence of both *C. immitis* and *C. posadasii*, providing evidence that both species can exist in the same geographic region (8). In contrast, only *C. posadasii* was found among clinical isolates from Colorado and Nevada. Phylogenetic analyses revealed that isolates from the same state do not form monophyletic clades, suggesting multiple independent introductions rather than a single expansion. We identified at least seven introductions of *C. posadasii* into Utah, two into Nevada, and seven into Colorado.

Our findings align with previous studies that identified significant phylogeographic structure within *Coccidioides* species (8, 15). *C. posadasii* exhibits substantial population differentiation, forming distinct phylogeographic clades, including Arizona and Texas/Mexico/South America clades, as well as a recently identified Guatemala/Venezuela clade. In contrast, *C. immitis* populations appear geographically restricted primarily to California, with a distinct, recently recognized clade in Washington (8, 15). However, despite the apparent structure of these clades, we observed substantial genetic variation within each clade, likely reflecting large effective population sizes (13). Consequently, isolates did not cluster into strictly state-specific monophyletic clades. Our phylogenetic analyses revealed that *C. posadasii* predominates in low-endemicity regions, particularly Utah, Colorado, and Nevada. The presence of multiple independent introductions into these states suggests that *Coccidioides* migration is not a singular event but an ongoing process. These migration events likely stem from high-endemicity regions such as Arizona and Texas, where *C. posadasii* maintains greater genetic diversity. Our ancestral area reconstruction supports these findings, indicating that multiple invasions into low-endemicity areas occurred independently from distinct ancestral populations.

The findings of this study have significant implications for public health. Underdiagnosis and misdiagnosis of coccidioidomycosis remain persistent challenges, particularly in regions where the disease is not typically considered by clinicians (16, 17). Our results underscore the urgent need for continued surveillance and increased awareness among healthcare professionals and the public in low-incidence states. The broad geographic distribution of *C. posadasii* isolates further highlights the value of genomic surveillance in tracking pathogen spread, monitoring evolutionary dynamics, and identifying emerging epidemiological patterns.

Travel history is a critical component in understanding the transmission dynamics of coccidioidomycosis. Previous genomic studies have shown that many cases diagnosed in non-endemic regions are linked to travel to endemic areas, emphasizing that infections can be acquired far from where symptoms eventually appear (4, 18). Our findings are consistent with this observation, for example, one patient’s *C. immitis* isolate was likely acquired in California, despite the diagnosis occurring elsewhere. This supports the need for clinicians to consider travel exposure during diagnosis. Moreover, the ability of genomic epidemiology to trace isolates back to their probable geographic origins demonstrates the power of integrating molecular surveillance with patient travel histories to improve diagnostic precision and guide public health interventions (4, 19). Strengthening these efforts is essential for tracking the spread of *Coccidioides* and anticipating shifts in disease distribution.

While our findings offer valuable insights, there are some limitations to consider. The genomic sampling strategy provides only a minimum estimate of the number of introductions into each location, as undetected lineages and uneven sampling across states may obscure the full extent of *Coccidioides* diversity and migration. Second, by sampling clinical isolates sent to a national diagnostic laboratory, we may exclude *Coccidioides* lineages that are associated with less severe symptoms or lineages that exist in enzootic cycles but are less infectious to humans (20).

Third, we were only able to collect travel histories for a subset of patients who were part of the University of Utah Health system, due to limits on protected health information associated with samples diagnosed at ARUP from other healthcare systems, diagnostic laboratories, and hospitals. Some *Coccidioides* isolates may reflect exposure during travel to endemic regions rather than local transmission (4). This is a known limitation of analyzing clinical isolates, as our location information reflects the location of diagnosis rather than the site of infection. Future research should expand genomic sampling across broader geographic and environmental ranges and collect detailed travel histories to clarify dispersal patterns and distinguish between locally acquired and travel-associated *Coccidioides* species.

Our study extends previous work by characterizing the genetic diversity and migration history of *Coccidioides* in underrepresented regions. We find that *C. posadasii* has independently colonized states with low incidence of Valley fever multiple times, consistent with a pattern of ongoing gene flow, rather than a single introduction. These findings emphasize the importance of ongoing genomic surveillance to monitor ongoing *Coccidioides* dispersal.

## MATERIALS AND METHODS

### Sample collection

We prospectively collected *Coccidioides*-positive isolates submitted to ARUP Laboratories, a national diagnostic laboratory from January 2023 to November 2024. We selected isolates from low-endemicity states, defined as those reporting fewer than 500 cases per year, that had limited available *Coccidioides* genomes, including 16 from Utah, 7 from Colorado, and 4 from Nevada (Fig. 1). We additionally included prospectively sampled isolates from California due to its well-documented high endemicity (CDC; https://www.cdc.gov/valley-fever/hcp/clinical-overview/) for *C. immitis* and relative lack of available whole-genome sequences. We additionally included 136 sequences from previously published studies to achieve broad genomic representation across diverse geographic regions (Fig. 1). A list of accession numbers, species assignments, and geographic origins for the previously published samples used in this study is available in Table S1.

### Whole-genome sequencing

Genomic DNA was extracted from fungal cultures at ARUP using a Maxwell RSC Cell DNA purification kit (AS1370). DNA samples (1–25 ng) were enzymatically fragmented, and libraries were prepared using the New England Biolabs NEBNext Ultra II FS DNA Library Prep kit (cat# E7805L) with an average insert size of 350 bp. PCR-amplified libraries were qualified on an Agilent Technologies 4150 TapeStation using a D1000 ScreenTape assay (cat# 5067-5582 and 5067-5583), and the molarity of adapter-modified molecules was defined by quantitative PCR using the Kapa Biosystems Kapa Library Quant Kit (cat# KK4824). Libraries were normalized and pooled in preparation for Illumina sequencing. We conducted paired-end whole-genome sequencing (150 × 2 bp) on an Illumina NovaSeq X Series at the University of Utah High-Throughput Sequencing Core.

### Variant calling

Variant calling was performed using cocci-call (21), a bioinformatics pipeline specifically developed for species identification and detection of single-nucleotide polymorphisms (SNPs) and other genomic variants in *Coccidioides*. Within cocci-call, raw sequencing reads were preprocessed by trimming low-quality bases (Phred < 20) and removing adapters using Trim Galore (stringency = 1) (https://github.com/FelixKrueger/TrimGalore). Quality control reports were generated with FastQC (https://www.bioinformatics.babraham.ac.uk/projects/fastqc/). To minimize false variant detection due to contamination, Kraken2 (22) was used to classify reads taxonomically, removing non-*Coccidioides* reads. Species assignment was based on the log2-transformed ratio of unique minimizers assigned to *C. immitis* or *C. posadasii*. Following this, high-quality reads were mapped to species-specific reference genomes (*C. immitis* GCF_000149335.2 and *C. posadasii* GCA_018416015.2) using BWA-MEM (v0.7.17) (23), and duplicate reads were marked with GATK 4.3 (24). If species assignment remained ambiguous, reads were mapped to both references, with assignment determined by the highest mapping percentage. To minimize the impact of repetitive elements on our data set, repetitive sequences were identified and removed using both NUCmer (25) and RepeatMasker (https://www.repeatmasker.org/). Variants were called using GATK HaplotypeCaller and GenotypeGVCFs, with sample ploidy set to 1. After variant calling, samples with a mean coverage lower than 30× were removed. Since the coalescent model assumes neutrality, we extracted only intergenic regions from the VCF using annotation data, processed with SnpEff (26). Finally, VCF files were converted to FASTA format using vcf2msa (available at https://github.com/mmarchetti90/vcf2msa), retaining only variant sites.

### Phylogenetic analysis

We employed a maximum likelihood approach to reconstruct the relationship of the sampled isolates using IQ-TREE 2 (27). The multiple sequence alignment file was generated using cocci-call, including only variant sites from intergenic regions. Model selection was performed with ModelFinder Plus (28), which evaluates multiple substitution models based on the Bayesian information criterion and incorporates ascertainment bias correction to account for the absence of invariant sites. To ensure robust statistical support for the tree topology, we assessed branch support using 1,000 replicates of ultrafast bootstrap approximation (29). The phylogenetic tree was rooted using the midpoint rooting method, which places the root at the midpoint of the longest path between any two taxa, providing an optimal approximation when an outgroup is not available.

### Ancestral area reconstruction

To infer the historical migration of *Coccidioides*, we used ancestral area reconstruction with the R package *ape* (30). Specifically, we utilized the function ace to estimate ancestral character states and their associated uncertainty. We considered locality as a discrete character, which we defined as the state of the submitting diagnostic lab for newly sequenced isolates from ARUP and the US state or country of origin for previously published sequence data. In total, we included 18 localities: 15 US states and 3 countries. We reconstructed the most probable historical dispersal routes and potential origins of specific lineages.

### Exposure histories

For patients who are diagnosed in Utah, we conducted retrospective chart reviews to collect information on symptoms, diagnosis, and exposure history, including any information recorded on possible occupational, recreational, or travel exposures. We additionally mailed letters to patients who tested positive for coccidioidomycosis in Utah, inviting them to participate in the study, with a link to a RedCap survey, which included information on their exposure history and clinical symptoms. Patients/participants of any age were recruited.

## Data Availability

The authors confirm that all data underlying the findings are fully available without restriction. The FASTA file used for phylogenetic analyses is publicly available at https://github.com/emanuelmfonseca/coccidioides-phylogenetic-data. All sequences, including those generated in this study and previously available data, have been deposited under BioProject accession number PRJNA1337116. The complete list of accession numbers for all samples used in this study is provided in the Supplemental material section.

## SUPPLEMENTAL MATERIAL

Table S1 spectrum.01822-25-s0001.pdf Accession numbers, species identification, and geographic origin for all *Coccidioides* genomes used in this study. Accession numbers correspond to entries in the NCBI Sequence Read Archive (SRA). Species designations (*Coccidioides immitis* or *Coccidioides posadasii*) were inferred using the cocci-call pipeline. Geographic origin refers to the state or country where the isolate was originally collected. When multiple locations are listed (e.g., “Washington/Oregon”), the exact origin could not be resolved beyond that level.

ASM does not own the copyrights to Supplemental Material that may be linked to, or accessed through, an article. The authors have granted ASM a non-exclusive, world-wide license to publish the Supplemental Material files. Please contact the corresponding author directly for reuse.
