# Supplementary material for: Coccidioides genomes from low-incidence states reveal complex migration history across the western United States
Source: Microbiol Spectr. 2025 Oct 24;13(12):e01822-25. doi: 10.1128/spectrum.01822-25 (PMC12671215; doi:10.1128/spectrum.01822-25)
Supplement: Table S1 — Accession numbers, species identification, and geographic origin for all Coccidioides genomes used in this study. Accession numbers correspond to entries in the NCBI Sequence Read Archive (SRA). Species designations (Coccidioides immitis or Coccidioides posadasii) were inferred using the cocci-call pipeline. Geographic origin refers to the state or country where the isolate was originally collected. When multiple locations are listed (e.g., “Washington/Oregon”), the exact origin could not be resolved beyond that level. [file spectrum.01822-25-s0001.pdf]

SUPPORTING INFORMATION

Coccidioides Genomes from Low-Incidence States Reveal Complex Migration History across the Western United States

Emanuel M. Fonseca[1,2]*, Shanaya Fox[1], Adrienne L. Carey[3], Bridget Barker[4], Marco Marchetti[5], Megan Hirschi[3], Kimberly E. Hanson[3],[6], Katharine S. Walter[1]

[1] Division of Epidemiology, University of Utah, Salt Lake City, Utah, USA

[2] Elfa Analytics, Ponte Nova, Minas Gerais, Brazil

[3] Department of Medicine, Division of Infectious Diseases, University of Utah School of Medicine, Salt Lake City, Utah, USA

[4] Department of Biological Sciences, Northern Arizona University, Flagstaff, Arizona, USA

[5] Eccles Institute of Human Genetics, University of Utah, Salt Lake City, Utah, USA

[6] Department of Pathology, Division of Clinical Microbiology, University of Utah and ARUP Laboratories, Salt Lake City, Utah, USA

*** Corresponding Author:** Emanuel M. Fonseca, emanuelmfonseca@gmail.com

Table S1. Accession numbers, species identification, and geographic origin for all Coccidioides genomes used in this study. Accession numbers correspond to entries in the NCBI Sequence Read Archive (SRA). Species designations (*Coccidioides immitis* or *Coccidioides posadasii*) were inferred using the cocci-call pipeline. Geographic origin refers to the state or country where the isolate was originally collected. When multiple locations are listed (e.g., "Washington/Oregon"), the exact origin could not be resolved beyond that level.

Accession Number	Species	Geographic Origin
SRS26766198	*Coccidioides immitis*	California
SRS26766197	*Coccidioides posadasii*	Utah
SRS26766196	*Coccidioides posadasii*	Utah
SRS26766195	*Coccidioides posadasii*	Utah
SRS26766194	*Coccidioides posadasii*	Utah
SRS26766193	*Coccidioides posadasii*	Utah
SRS26766192	*Coccidioides posadasii*	Nevada
SRS26766191	*Coccidioides posadasii*	Nevada
SRS26766190	*Coccidioides posadasii*	Nevada
SRS26766189	*Coccidioides immitis*	California
SRS26766188	*Coccidioides posadasii*	Nevada
SRS26766187	*Coccidioides posadasii*	Colorado
SRS26766186	*Coccidioides posadasii*	Colorado
SRS26766185	*Coccidioides posadasii*	Colorado
SRS26766184	*Coccidioides posadasii*	Colorado
SRS26766183	*Coccidioides posadasii*	Colorado
SRS26766182	*Coccidioides posadasii*	Colorado
SRS26766181	*Coccidioides immitis*	California
SRS26766180	*Coccidioides immitis*	California
SRS26766179	*Coccidioides immitis*	California
SRS26766178	*Coccidioides immitis*	California
SRS26766177	*Coccidioides posadasii*	Utah
SRS26766176	*Coccidioides posadasii*	Utah
SRS26766175	*Coccidioides posadasii*	Utah
SRS26766174	*Coccidioides immitis*	Utah
SRS26766173	*Coccidioides immitis*	Utah
SRS26766172	*Coccidioides immitis*	Utah
SRS26766171	*Coccidioides posadasii*	Utah
SRS26766170	*Coccidioides posadasii*	Utah

SRS26766169	*Coccidioides posadasii*	Utah
SRS26766168	*Coccidioides immitis*	California
SRS26766167	*Coccidioides posadasii*	Utah
SRS26766166	*Coccidioides posadasii*	Utah
SRS26766165	*Coccidioides posadasii*	Colorado
SRS26766164	*Coccidioides immitis*	California
SRS26766163	*Coccidioides posadasii*	California
SRS26766162	*Coccidioides immitis*	California
SRS26766161	*Coccidioides immitis*	California
SRS26766160	*Coccidioides immitis*	California
SRS26766159	*Coccidioides immitis*	California
SRS26766158	*Coccidioides immitis*	California
SRS26766157	*Coccidioides immitis*	California
SRS26766156	*Coccidioides immitis*	California
SRS26766155	*Coccidioides immitis*	California
SRS26766154	*Coccidioides immitis*	California
SRS26766153	*Coccidioides immitis*	California
SRS26766152	*Coccidioides posadasii*	California
SRS26766151	*Coccidioides immitis*	California
SRS26766150	*Coccidioides immitis*	California
SRR1292218	*Coccidioides immitis*	California
SRR1292219	*Coccidioides immitis*	California
SRR1292224	*Coccidioides immitis*	Washington
SRR1292225	*Coccidioides immitis*	Washington
SRR1292226	*Coccidioides immitis*	Washington
SRR1292227	*Coccidioides immitis*	Washington
SRR1292228	*Coccidioides immitis*	Washington
SRR21204671	*Coccidioides posadasii*	Michigan
SRR21204672	*Coccidioides posadasii*	Wisconsin
SRR21204673	*Coccidioides posadasii*	Minnesota
SRR21204674	*Coccidioides immitis*	Washington
SRR21204675	*Coccidioides immitis*	Washington
SRR21204676	*Coccidioides posadasii*	Oregon
SRR21204677	*Coccidioides immitis*	Washington
SRR21204678	*Coccidioides posadasii*	Washington/Oregon
SRR21204679	*Coccidioides immitis*	Oregon
SRR21204680	*Coccidioides immitis*	Oregon
SRR21204681	*Coccidioides posadasii*	Washington
SRR21204682	*Coccidioides immitis*	Michigan
SRR21204683	*Coccidioides immitis*	Oregon

SRR21204684	*Coccidioides immitis*	Maryland
SRR21204685	*Coccidioides posadasii*	Oregon
SRR21204686	*Coccidioides posadasii*	Washington
SRR21204687	*Coccidioides immitis*	Oregon
SRR21204688	*Coccidioides posadasii*	Washington
SRR21204689	*Coccidioides immitis*	Washington
SRR21204691	*Coccidioides immitis*	Washington
SRR21204692	*Coccidioides immitis*	Oregon
SRR21204693	*Coccidioides immitis*	Oregon
SRR21204694	*Coccidioides immitis*	Oregon
SRR21204695	*Coccidioides immitis*	Washington
SRR21204696	*Coccidioides posadasii*	Oregon
SRR21204698	*Coccidioides immitis*	Washington
SRR21204699	*Coccidioides immitis*	Oregon
SRR21204700	*Coccidioides immitis*	Washington
SRR21204701	*Coccidioides immitis*	Oregon
SRR21204702	*Coccidioides posadasii*	Oregon
SRR21204703	*Coccidioides immitis*	Oregon
SRR21204704	*Coccidioides immitis*	Oregon
SRR21204705	*Coccidioides immitis*	Oregon
SRR21204706	*Coccidioides posadasii*	Oregon
SRR21204707	*Coccidioides posadasii*	Minnesota
SRR21204708	*Coccidioides posadasii*	Washington
SRR21204709	*Coccidioides immitis*	Washington
SRR21204710	*Coccidioides posadasii*	Washington
SRR21204711	*Coccidioides posadasii*	Washington
SRR21204713	*Coccidioides immitis*	Oregon
SRR21204714	*Coccidioides posadasii*	Oregon
SRR21204715	*Coccidioides posadasii*	Michigan
SRR21204716	*Coccidioides posadasii*	Michigan
SRR21204717	*Coccidioides posadasii*	Michigan
SRR21204718	*Coccidioides posadasii*	Michigan
SRR21204719	*Coccidioides posadasii*	Michigan
SRR21204720	*Coccidioides immitis*	Washington
SRR21204721	*Coccidioides posadasii*	Oregon
SRR21204722	*Coccidioides posadasii*	Michigan
SRR21204723	*Coccidioides immitis*	Oregon
SRR21204724	*Coccidioides immitis*	Wisconsin
SRR21204725	*Coccidioides posadasii*	Oregon
SRR21204726	*Coccidioides posadasii*	Michigan

SRR21204727	*Coccidioides posadasii*	Michigan
SRR21204728	*Coccidioides posadasii*	Michigan
SRR21204729	*Coccidioides posadasii*	Pennsylvania
SRR21204730	*Coccidioides posadasii*	Wisconsin
SRR21204731	*Coccidioides immitis*	Washington
SRR21204732	*Coccidioides posadasii*	New Mexico
SRR21292480	*Coccidioides posadasii*	Washington
SRR21292481	*Coccidioides posadasii*	Washington
SRR21292482	*Coccidioides posadasii*	Washington
SRR21292483	*Coccidioides posadasii*	Washington
SRR21295062	*Coccidioides posadasii*	Oregon
SRR21295063	*Coccidioides immitis*	Oregon
SRR21295064	*Coccidioides posadasii*	Oregon
SRR21295065	*Coccidioides posadasii*	Oregon
SRR21295066	*Coccidioides posadasii*	Oregon
SRR21295068	*Coccidioides posadasii*	Washington
SRR3468016	*Coccidioides immitis*	California
SRR3468019	*Coccidioides immitis*	California
SRR3468021	*Coccidioides immitis*	Mexico
SRR3468023	*Coccidioides posadasii*	Arizona
SRR3468024	*Coccidioides posadasii*	Arizona
SRR3468025	*Coccidioides posadasii*	Arizona
SRR3468028	*Coccidioides posadasii*	Arizona
SRR3468029	*Coccidioides posadasii*	Arizona
SRR3468030	*Coccidioides posadasii*	Arizona
SRR3468031	*Coccidioides posadasii*	Arizona
SRR3468032	*Coccidioides posadasii*	Arizona
SRR3468033	*Coccidioides posadasii*	Arizona
SRR3468034	*Coccidioides posadasii*	Arizona
SRR3468035	*Coccidioides posadasii*	Arizona
SRR3468036	*Coccidioides posadasii*	Arizona
SRR3468038	*Coccidioides immitis*	California
SRR3468040	*Coccidioides posadasii*	Arizona
SRR3468041	*Coccidioides posadasii*	Arizona
SRR3468043	*Coccidioides posadasii*	Arizona
SRR3468044	*Coccidioides posadasii*	Arizona
SRR3468045	*Coccidioides posadasii*	Arizona
SRR3468047	*Coccidioides posadasii*	Arizona
SRR3468048	*Coccidioides posadasii*	Texas
SRR3468049	*Coccidioides immitis*	California

SRR3468050	*Coccidioides posadasii*	Colorado
SRR3468051	*Coccidioides posadasii*	Mexico
SRR3468053	*Coccidioides posadasii*	Mexico
SRR3468055	*Coccidioides posadasii*	Arizona
SRR3468056	*Coccidioides posadasii*	Arizona
SRR3468057	*Coccidioides posadasii*	Arizona
SRR3468058	*Coccidioides posadasii*	Arizona
SRR3468061	*Coccidioides posadasii*	Arizona
SRR3468062	*Coccidioides posadasii*	Arizona
SRR3468063	*Coccidioides posadasii*	Arizona
SRR3468064	*Coccidioides posadasii*	Mexico
SRR3468065	*Coccidioides posadasii*	Mexico
SRR3468066	*Coccidioides posadasii*	Mexico
SRR3468067	*Coccidioides posadasii*	Guatemala
SRR3468069	*Coccidioides posadasii*	Texas
SRR3468070	*Coccidioides posadasii*	Guatemala
SRR3468071	*Coccidioides immitis*	California
SRR3468073	*Coccidioides posadasii*	Arizona
SRR3468075	*Coccidioides posadasii*	Texas
SRR3468081	*Coccidioides immitis*	California
SRR6830881	*Coccidioides posadasii*	Venezuela
SRR6830882	*Coccidioides posadasii*	Venezuela
SRR6830884	*Coccidioides posadasii*	Venezuela
SRR6830886	*Coccidioides posadasii*	Venezuela
SRR6830887	*Coccidioides posadasii*	Mexico
SRR6830888	*Coccidioides posadasii*	Venezuela
SRR7206598	*Coccidioides immitis*	Washington
SRR7206599	*Coccidioides immitis*	Washington
SRR7206601	*Coccidioides immitis*	Washington
SRR7206603	*Coccidioides immitis*	Washington
SRR7206605	*Coccidioides immitis*	Washington
SRR8530933	*Coccidioides immitis*	Washington
SRR8530934	*Coccidioides immitis*	Washington
SRR8530935	*Coccidioides immitis*	Washington
SRR8530936	*Coccidioides immitis*	Washington
SRR8530937	*Coccidioides immitis*	Washington
